# Distinct Spatiotemporal Distribution of Bacterial Toxin-Produced Cellular cAMP Differentially Inhibits Opsonophagocytic Signaling

**DOI:** 10.3390/toxins11060362

**Published:** 2019-06-20

**Authors:** Shakir Hasan, Waheed Ur Rahman, Peter Sebo, Radim Osicka

**Affiliations:** Institute of Microbiology of the CAS, v. v. i., Videnska 1083, 142 20 Prague, Czech Republic; shasan@uliege.be or shakirhasan@gmail.com (S.H.); waheed.rahman@biomed.cas.cz (W.U.R.); sebo@biomed.cas.cz (P.S.)

**Keywords:** 3′,5′-cyclic adenosine monophosphate (cAMP), adenylate cyclase toxin, edema toxin, opsonophagocytosis, phagocytes, Syk, Vav, Pyk2, signaling pathway

## Abstract

Myeloid phagocytes have evolved to rapidly recognize invading pathogens and clear them through opsonophagocytic killing. The adenylate cyclase toxin (CyaA) of *Bordetella pertussis* and the edema toxin (ET) of *Bacillus anthracis* are both calmodulin-activated toxins with adenylyl cyclase activity that invade host cells and massively increase the cellular concentrations of a key second messenger molecule, 3’,5’-cyclic adenosine monophosphate (cAMP). However, the two toxins differ in the kinetics and mode of cell entry and generate different cAMP concentration gradients within the cell. While CyaA rapidly penetrates cells directly across their plasma membrane, the cellular entry of ET depends on receptor-mediated endocytosis and translocation of the enzymatic subunit across the endosomal membrane. We show that CyaA-generated membrane-proximal cAMP gradient strongly inhibits the activation and phosphorylation of Syk, Vav, and Pyk2, thus inhibiting opsonophagocytosis. By contrast, at similar overall cellular cAMP levels, the ET-generated perinuclear cAMP gradient poorly inhibits the activation and phosphorylation of these signaling proteins. Hence, differences in spatiotemporal distribution of cAMP produced by the two adenylyl cyclase toxins differentially affect the opsonophagocytic signaling in myeloid phagocytes.

## 1. Introduction

Phagocytosis is a central event in the innate immune response and is defined as the mechanism for recognition and internalization of particles larger than 0.5 µm in diameter [1,2]. It is an evolutionarily conserved immune mechanism that is crucial for the first line of defense against pathogenic microbes and orchestrates adaptive immunity through antigen presentation. Professional phagocytes arrive at sites of infection, ingest invading pathogens, and mediate the clearance of the microbial infection [1,2].

Monocytes, macrophages, neutrophils, and dendritic cells express a variety of receptors that mediate (opsono)phagocytosis. Type I phagocytosis occurs through the Fcγ receptor (FcγR) that binds particles opsonized by antibodies. This triggers signal transduction through small guanosine triphosphatases (GTPases) of the Rho family, Cdc42, and Rac, leading to engulfment of the opsonized particle through protruding lamellopodia [3,4,5]. Type II phagocytosis is mediated by the complement receptor 3 (CR3; CD11b/CD18, α_M_β_2_, Mac-1), which recognizes particles coated with the opsonizing proteins of the complement system and triggers signal transduction through RhoA, leading to the sinking of the opsonized particle into the cell [3,4,5]. CR3 and other cellular receptors, such as the mannose receptor, dectin-1, Toll-like receptors, scavenger receptors, or CD14, recognize various microbial ligands and mediate non-opsonic phagocytosis of infecting microbes [6,7]. Phagocytosis involves activation of signaling mediators, such as the spleen tyrosine kinase (Syk) [8,9], the proline-rich tyrosine kinase 2 (Pyk2) [10,11], and the Rho family guanine nucleotide exchange factor Vav [12,13,14], which transduce the signal from the phagocytic receptors, provoking the uptake and subsequent phagocytic killing of invading pathogens.

Many bacterial pathogens secrete exotoxins that inhibit the bactericidal activity of phagocytes by specifically targeting the signaling pathways involved in the uptake of invading pathogens. A prominent class of such virulence factors are the toxins with adenylyl cyclase (AC) enzyme activity [15,16,17,18]. These toxins target and invade cells to elevate the cytosolic concentration of a key second messenger molecule, 3’,5’-cyclic adenosine monophosphate (cAMP), thereby provoking suppression of immune functions [16,19,20,21,22,23,24]. cAMP levels are then sensed by the protein kinase A (PKA), the exchange protein directly activated by cAMP (Epac) and by the cyclic nucleotide-gated ion channels, thereby modulating the activity of downstream signal transduction pathways and regulating cellular responses [25].

The gram-negative respiratory pathogen *Bordetella pertussis* secretes an adenylate cyclase toxin (CyaA) [18], which is a multifunctional RTX (repeats in toxin) toxin with a cell-invasive AC domain linked to a hemolysin moiety [26]. CyaA binds the integrin CR3 on myeloid phagocytes and translocates its catalytic domain directly across the plasma membrane of cells in a two-step process that is accompanied by influx of extracellular calcium ions into cells [27]. The half-time of AC translocation is about 30 s from cell contact [28] and inside the cytosol the AC enzyme domain is activated by calmodulin to catalyze unregulated conversion of cellular ATP into cAMP [26,29]. In parallel, the hemolysin moiety of CyaA forms oligomeric pores in the plasma membrane of cells and mediates K^+^ efflux from cells [29,30].

A structurally homologous AC enzyme is produced by the Gram-positive zoonotic pathogen *Bacillus anthracis* that secretes an A-B type edema toxin (ET) consisting of protective antigen (PA) and edema factor (EF) [15]. PA uses the tumor endothelial marker 8 (TEM8, ANTXR1) and the capillary morphogenesis gene 2 protein (CMG2, ANTXR2) as receptors [31]. Upon cell binding, PA is processed by furin-like proteases and oligomerizes on the cell surface to form heptamers or octamers. EF, the enzymatic subunit, binds PA oligomers and the formed EF-PA complex is internalized into early endosomes via lipid raft-mediated clathrin-dependent endocytosis [32]. Within 30 min of cell contact, the EF translocates across the endosomal membrane, through a channel formed by PA (triggered by low pH due to acidification) and reaches cell cytosol, where it is activated by calcium-loaded calmodulin and catalyzes conversion of ATP into cAMP [15,31].

The kinetics and mode of entry of CyaA and ET into target cells differ, thus yielding cAMP production in different zones of the cell cytosol [33]. CyaA delivers its AC domain directly into the submembranous zone of cell cytoplasm, without the need for endocytosis and thus rapidly starts to intoxicate the cells. This results in a membrane proximal pool of cAMP that forms a gradient that decreases from the membrane towards the perinuclear region of the cell. In contrast, ET has to be endocytosed with the receptor and its catalytic subunit translocates across the endosomal membrane to its cytosolic side, where the AC enzyme (EF) remains attached and catalyzes production of cAMP. This results in a substantial time lag before a cAMP concentration gradient starts to form, decreasing from the perinuclear area towards the submembranous zone of the cytosol [33].

It was previously shown that CyaA-mediated cAMP signaling swiftly inhibits the bactericidal activity of phagocytes by modulating the activity of key components of the signaling cascades involved, such as Syk, RhoA, and SHP-1 [26,29,34,35,36]. Here we show that differences in cellular cAMP concentration gradients produced by CyaA and ET result in different inhibitory effects on the signaling cascades that drive opsonophagocytosis in human myeloid phagocytes.

## 2. Results

### 2.1. CyaA and ET Differ in the Temporal Characteristics of cAMP Intoxication of Human Monocytes

**Figure 1:**
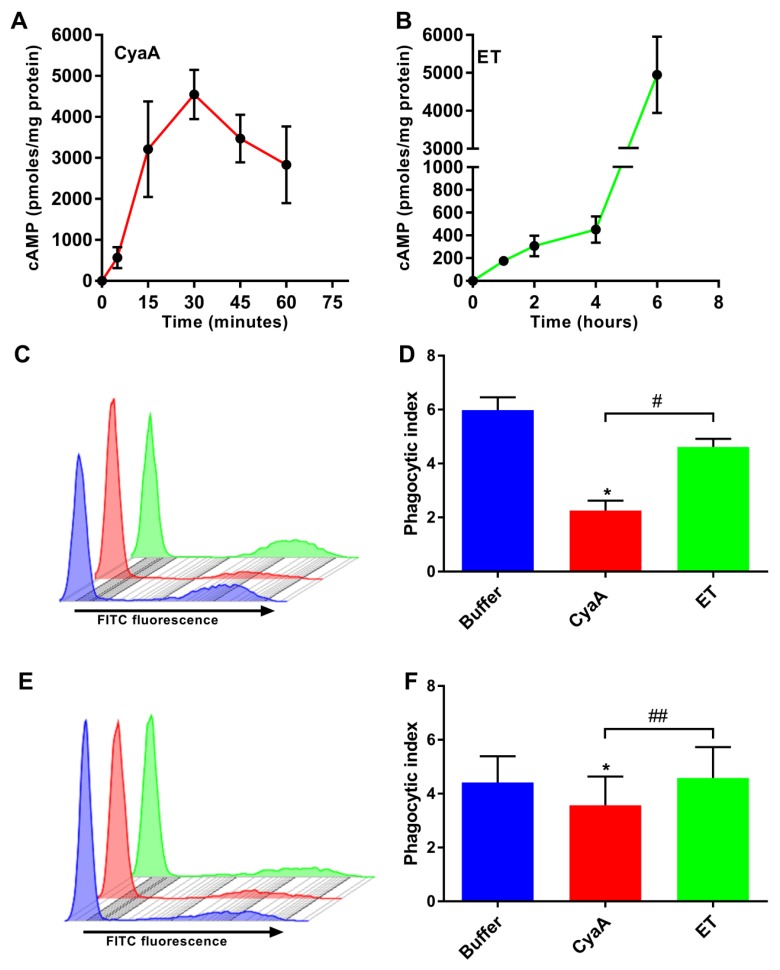
Adenylate cyclase toxin (CyaA)- but not edema toxin (ET)-provoked 3’,5’-cyclic adenosine monophosphate (cAMP) elevation leads to inhibition of uptake of fluorescently labelled serum-opsonized zymosan (SOZ) particles. (**A** and **B**) THP-1 human monocytes were incubated with CyaA (A) or ET (B) for the indicated times. Cellular cAMP levels were determined using competitive ELISA and normalized to cellular protein concentration. (**C** and **D**) 2 × 10^5^ THP-1 cells were preincubated with buffer (blue), CyaA for 30 min (red), or ET for 6 h (green) as mentioned in the Materials and Methods section. The cells were washed and incubated with fluorescently labelled SOZ particles for 30 min, before binding and internalization of fluorescent particles were analyzed using flow cytometry. Representative histogram from one experiment (C) and the calculated phagocytic index (**D**) are shown. Data represent mean with SEM (N = 3). P values were determined by paired one-way ANOVA; *, *p* < 0.05 for the results compared with buffer-treated cells; #, *p* < 0.05 for results comparing CyaA- and ET-treated cells. (**E** and **F**) Bacterial toxin and fluorescently labelled SOZ particles were added simultaneously to the cells and processed as mentioned in the legend to panels C and D. Representative histogram from one experiment (E) and the calculated phagocytic index (F) are shown. The phagocytic index was calculated by data extracted out of flow cytometry experiments as outlined in detail in the Materials and Methods section. Data represent mean with SEM (N = 4). *p* values were determined using paired one-way ANOVA; *, *p* < 0.05 for the results compared with buffer-treated cells; ##, *p* < 0.005 for results comparing CyaA and ET-treated cells.

We first compared the time course of cAMP production in THP-1 cells exposed to CyaA or ET, in order to define a time point at which comparable overall levels of cellular cAMP were reached. Towards this aim, the toxins were incubated with human THP-1 monocytes that express the cellular receptors for both toxins [26,37,38]. As shown in Figure 1A, CyaA (150 ng/ml; 0.85 nM) rapidly elevated cellular cAMP levels in THP-1 monocytes and cAMP accumulation was already detectable at five minutes after addition of the toxin, reaching a plateau at 30 min of incubation.

In contrast, even at a higher molar concentration, ET (625 ng/ml; 7.04 nM EF + 2500 ng/ml; 30.24 nM PA) triggered a delayed and relatively slow rise of cAMP levels over the initial four hours, followed by a rapid increase of cAMP level (Figure 1B). This corresponded to the time taken for internalization of the ET-receptor complex and subsequent translocation of EF across the membrane of acidified endosomes [31,33]. Similar cAMP intoxication trends were previously reported for T-cells [39]. Thus, in all further experiments, THP-1 monocytes were treated with CyaA (150 ng/ml; 0.85 nM) for 30 min or ET (625 ng/ml; 7.04 nM EF + 2500 ng/ml; 30.24 nM PA) for 6 h, in order to obtain cells intoxicated with similar overall levels of cellular cAMP.

### 2.2. Toxin-Provoked cAMP Signaling Inhibits Phagocytosis of Opsonized Targets

CyaA- and ET-provoked cAMP elevation hijacks signal transduction pathways and interferes with the opsonophagocytosis and bactericidal activities of phagocytes [16,20,22,34,35,36,40]. We thus used flow cytometry to compare the effects of CyaA and ET action (at similar overall cellular cAMP levels) on binding and internalization of serum-opsonized zymosan (SOZ) particles by THP-1 monocytes. As shown in Figure 1, incubation of THP-1 cells with CyaA for 30 min yielded ~5000 pmol of cAMP per mg of total cell protein (Figure 1A) and resulted in strong inhibition of phagocytic uptake of fluorescent SOZ particles by the cells (Figure 1C), which is reflected by the calculated phagocytic index (Figure 1D). In contrast, incubation of THP-1 cells with ET for 6 h, which also yielded ~5000 pmol of cAMP per mg of total cell protein (Figure 1B), had no significant effect on the phagocytic capacity of the cells (Figure 1C), which is reflected in the calculated phagocytic index (Figure 1D). Furthermore, when CyaA and SOZ particles were added simultaneously to THP-1 cells, the CyaA toxin was able to significantly inhibit the binding and uptake of fluorescently labeled SOZ particles (Figure 1E,F). In contrast, ET had no effect when added simultaneously with SOZ (Figure 1E,F). These results show that cAMP intoxication of THP-1 cells by CyaA occurs rapidly and efficiently inhibits opsonophagocytosis, while ET-provoked cAMP intoxication occurs gradually and is inefficient at inhibiting the binding and uptake of opsonized targets.

### 2.3. Toxin-Provoked cAMP Signaling Interferes with Signaling Downstream of Opsonin Receptor

**Figure 2:**
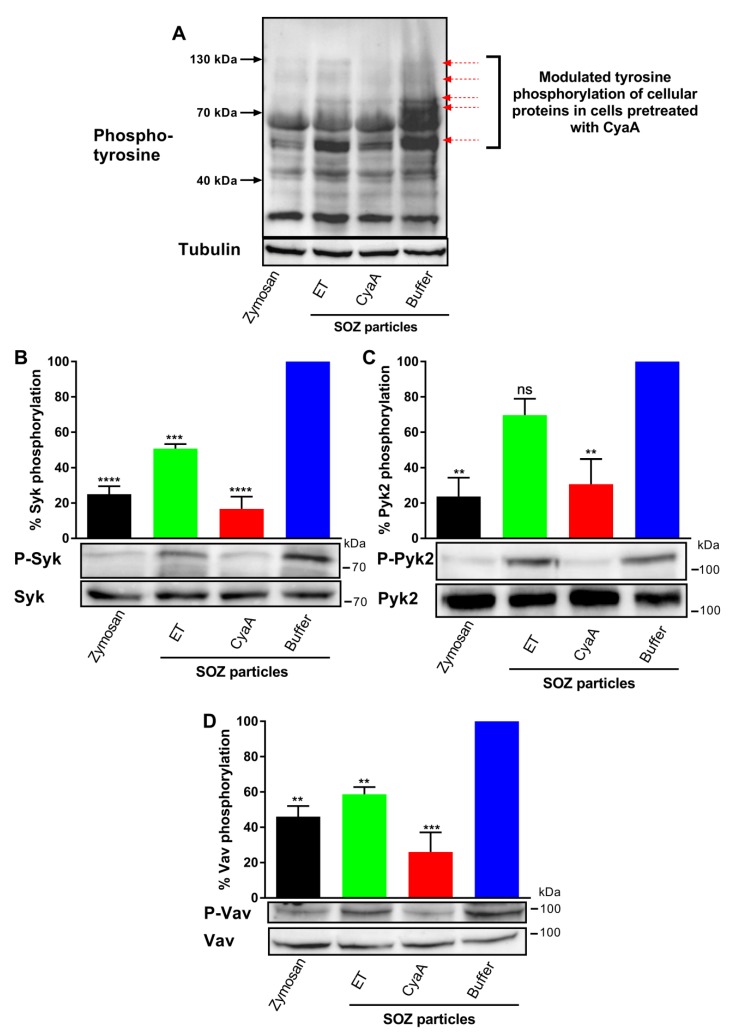
Toxin-provoked cAMP accumulation inhibits opsonin-induced tyrosine phosphorylation of cellular proteins Syk, Pyk2, and Vav. 3 × 10^6^ THP-1 human monocytes were preincubated with ET for 6 h (green), CyaA for 30 min (red), or buffer (blue) and subsequently incubated with SOZ particles (30 min) at 37 °C to induce tyrosine phosphorylation of crucial signaling proteins leading to opsonophagocytosis. THP-1 cells preincubated with buffer and then treated with unopsonized zymosan were taken as negative control (black). Cell lysates were analyzed by immunoblotting. (**A**) Modulated SOZ-induced tyrosine phosphorylation of proteins was detected in cellular lysates from toxin/buffer pretreated cells (red arrows); tubulin was used as loading control. Tyrosine phosphorylation of Syk (**B**), Pyk2 (**C**), and Vav (**D**) was detected using phospho-specific antibodies. Immunoblots developed with anti-Syk, anti-Pyk2, and anti-Vav antibodies served as loading controls. Data represent mean with SEM (N = 3). *p* values were determined using one-way ANOVA; **, *p* < 0.005; *** *p* < 0.001; **** *p* < 0.0001; ns, not significant, for results compared with buffer-treated cells incubated with SOZ particles.

To trigger the opsonin receptor-activated signaling pathways involved in opsonophagocytosis [8,36], THP-1 cells were incubated with SOZ particles and non-opsonized zymosan particles were used as a control. Figure 2A shows that SOZ particles induced phosphorylation of tyrosine residues of several cellular proteins, indicating the recruitment of non-receptor protein tyrosine kinases or a change in protein tyrosine phosphatase activity [41]. While preincubation with ET had little effect, preincubation of cells with CyaA led to much stronger inhibition of SOZ-induced tyrosine phosphorylation of the proteins. Hence, at similar cellular cAMP levels, the previously observed differences in CyaA and ET-produced spatial gradients of cAMP within the cell cytoplasm [33] differentially affected cAMP signaling-mediated inhibition of the signaling cascades that drive the opsonophagocytic uptake of particles.

### 2.4. CyaA-Provoked cAMP Signaling Inhibits Opsonin-Triggered Phosphorylation of Syk, Pyk2, and Vav

Intracellular signaling mediators Syk, Pyk2, and Vav are crucial for signal transduction during phagocytosis of opsonized targets [1,2]. Given that CyaA hampered opsonophagocytosis, we examined the inhibitory effects of toxin-provoked cAMP elevation on the phosphorylation of tyrosine residues of Syk (Y525/Y526), Pyk2 (Y402), and Vav (Y174) proteins.

The non-receptor tyrosine kinase Syk is recruited by the immunoreceptor tyrosine-based activation motifs (ITAMs) and is phosphorylated (activated) in the course of opsonophagocytosis, forming a multi-molecular signaling complex in which Syk-mediated phosphorylation of several adaptor proteins promotes activation of key signal transduction pathways [8,9,11]. As shown in Figure 2B, incubation of THP-1 cells with SOZ particles triggered tyrosine phosphorylation of Syk, indicating the activation of signaling pathways involved in opsonophagocytosis. Preincubation of cells with CyaA inhibited the phosphorylation and activation of Syk kinase (Figure 2B), while preincubation of cells with ET, at similar cellular cAMP levels, resulted only in partial inhibition (~ 50%) of Syk phosphorylation.

To corroborate this observation, we next examined the activation of the cytoplasmic non-receptor tyrosine kinase Pyk2, which is activated by signaling through phagocytic receptors or upon increase of cytosolic calcium concentration. Pyk2 activation is downstream to activation of the Syk and Src kinases and regulates the Rho/WASP pathway of actin-polymerization [10,11,42,43]. In line with the trend observed for Syk, preincubation of THP-1 cells with CyaA led to near complete inhibition of Pyk2 phosphorylation in cells incubated with SOZ particles (Figure 2C), whereas preincubation of cells with ET yielded only a modest inhibition of Pyk2 phosphorylation, which was not statistically significant.

Finally, we corroborated the above observations by examining activation of the Rho family guanine nucleotide exchange factor Vav, which plays a crucial role in the transmission of signals from the phagocytic receptors to Rho GTPases during phagocytosis. Syk physically interacts with Vav downstream of ITAMs and phosphorylates Vav on tyrosine-174 [8,12,14,44]. We therefore analyzed the effects of toxin-triggered cAMP signaling on tyrosine phosphorylation of Vav. Indeed, THP-1 cell incubation with SOZ particles induced tyrosine phosphorylation of Vav, and this was completely inhibited in cells that were preincubated with CyaA (Figure 2D). In contrast, preincubation of cells with ET led to only partial inhibition (~50%) of Vav phosphorylation.

Hence, CyaA-produced membrane-proximal cAMP intoxication [33] in monocytes led to signaling events that completely inhibited tyrosine phosphorylation of crucial signaling proteins Syk, Pyk2, and Vav involved in phagocytosis. In contrast, at equal overall cAMP concentrations in the cells, ET-produced cAMP intoxication in the perinuclear region affected the tyrosine phosphorylation of these crucial signaling proteins only mildly.

### 2.5. A Pharmacological Inhibitor of AC Enzyme Activity Reverses CyaA-Mediated Inhibition of Tyrosine Phosphorylation

**Figure 3:**
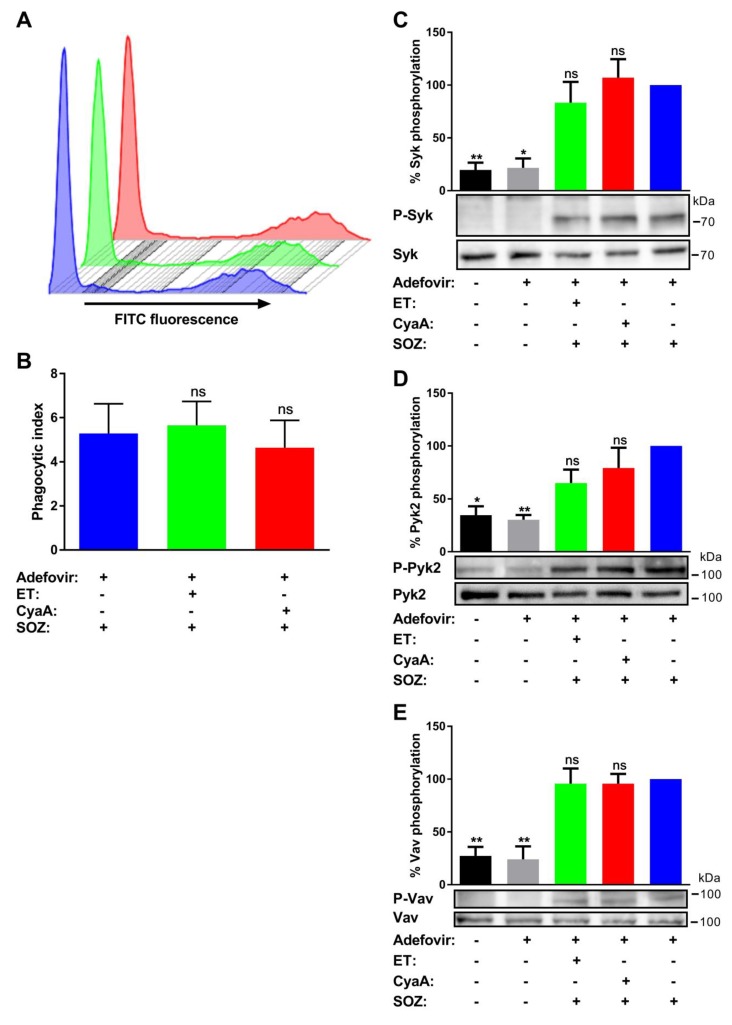
Cell permeable pharmacological inhibitor of AC enzyme activity reverses toxin-mediated inhibition of uptake of opsonized particles and associated signal transduction pathways. THP-1 human monocytes were preincubated with adefovir dipivoxil (10 µM) for 6 h, followed by incubation with ET for 6 h (green), CyaA for 30 min (red), or buffer (blue) as mentioned in the Materials and Methods section. (**A** and **B**) 2 × 10^5^ THP-1 cells were incubated with fluorescently labelled SOZ particles for 30 min, and binding and internalization of fluorescent particles were analyzed by flow cytometry. Representative histogram from one experiment (**A**) and the calculated phagocytic index (**B**) are shown. Data represent mean with SEM (N = 4). *p* values were determined by paired one-way ANOVA; ns, not significant for results compared with adefovir-treated cells incubated with SOZ particles. (**C** to **E**) 3 × 10^6^ THP-1 cells were incubated with SOZ particles to induce tyrosine phosphorylation of crucial signaling proteins involved in opsonophagocytosis. The cells were lysed, and the lysates were used for immunoblotting. Tyrosine phosphorylation of Syk (**C**), Pyk2 (**D**), and Vav (**E**) were detected using phospho-specific antibodies. Immunoblots developed using anti-Syk, anti-Pyk2, and anti-Vav antibodies served as loading controls. THP-1 cells preincubated without (black) or with (grey) adefovir and then treated with unopsonized zymosan were used as negative controls. Data represent mean with SEM (N = 3). *p* values were determined using one-way ANOVA; *, *p* < 0.05; **, *p* < 0.005; ns, not significant, for results compared with adefovir-treated cells incubated with SOZ particles.

Adefovir diphosphate, a cellular metabolite of the clinically approved pro-drug adefovir dipivoxil, was shown to competitively and selectively inhibit the AC enzyme activities of EF (Ki = 27 nM) and of CyaA (Ki = 25 nM) [45]. Therefore, we pretreated THP-1 monocytes with adefovir dipivoxil, prior to addition of the bacterial AC toxins, and SOZ particles were subsequently added to induce signaling through the pathways that govern the uptake of opsonized particles. Pretreatment of cells with adefovir dipivoxil reversed CyaA-mediated inhibition of uptake of fluorescently labeled SOZ particles (Figure 3A; cf Figure 1C) and yielded a calculated phagocytic index similar to cells treated with buffer or ET (Figure 3B).

**Figure A1:**
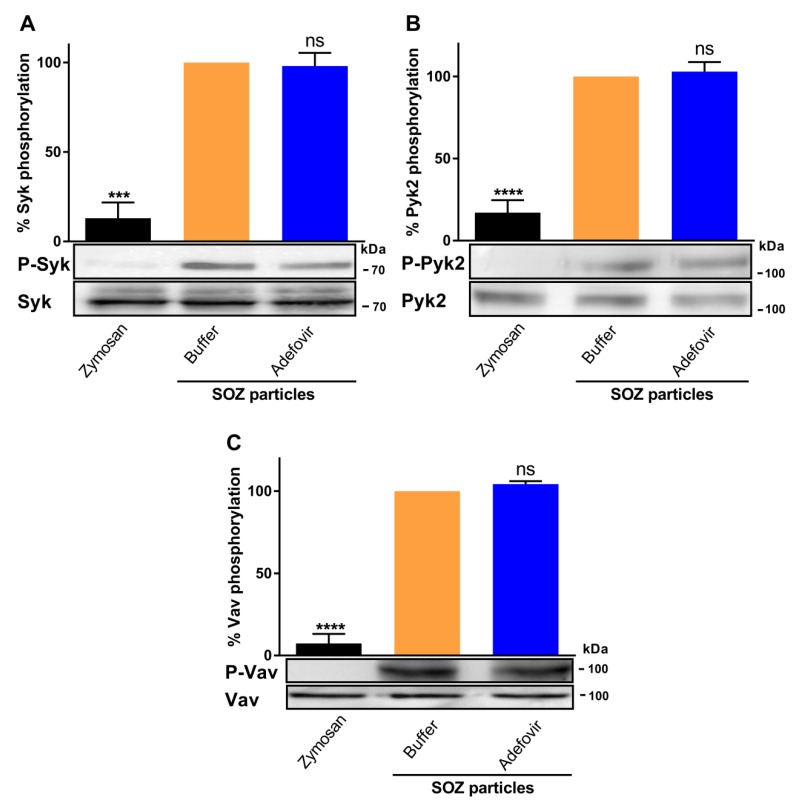
Preincubation of THP-1 cells with adefovir dipivoxil does not affect the opsonin induced tyrosine phosphorylation of Syk, Pyk2, and Vav. The experiment was carried out as mentioned in the legend for Figure 3. Data represent mean with SEM (N = 3). *p* values were determined by one-way ANOVA; *** *p* < 0.001; **** *p* < 0.0001; ns, not significant, for results compared with buffer-treated cells incubated with SOZ particles.

At the same time, adefovir dipivoxil itself did not trigger any changes in tyrosine phosphorylation of Syk, Pyk2, and Vav (Figure 3C–E) or influence the SOZ-triggered tyrosine phosphorylation of these signaling proteins (Figure A1). Furthermore, adefovir pretreatment also rescued the SOZ-induced phosphorylation of Syk (Figure 3C), Pyk2 (Figure 3D), and Vav (Figure 3E) in toxin-treated cells. These results suggest that the inhibition of AC enzyme activity reversed CyaA toxin-mediated inhibition of SOZ-induced phosphorylation of crucial signaling proteins. Furthermore, it also suggests that in the absence of AC enzyme activity, other CyaA toxin associated activities, such as Ca^2+^ influx, K^+^ efflux, and pore formation in the cellular membrane, do not affect the SOZ-induced tyrosine phosphorylation of Syk, Pyk2, and Vav.

### 2.6. CyaA- and ET-Provoked cAMP Signaling Differentially Affects Actin Cytoskeleton Remodeling in Phagocytes

**Figure 4:**
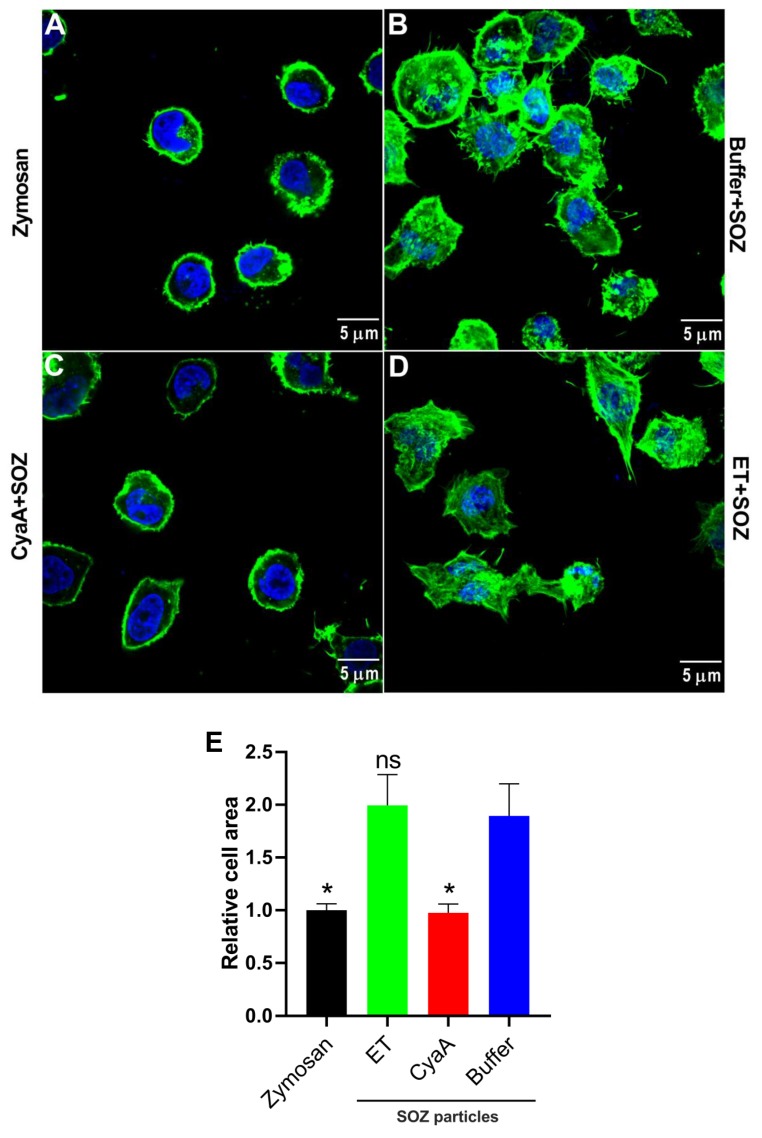
CyaA- and ET-provoked cAMP signaling differentially affects actin cytoskeleton remodeling in THP-1 monocytes. 3 × 10^6^ THP-1 cells were seeded on glass cover slips and incubated with unopsonized zymosan particles (**A**), or after preincubation with buffer (**B**), CyaA for 30 min (**C**), or ET for 6 h (**D**) with SOZ particles (30 min, 37 °C). After fixation, permeabilization, and staining for F-actin with TRITC-conjugated phalloidin (green) and for nucleic acid with DAPI (blue), the cells were imaged using confocal microscopy. All the images were processed using ImageJ software and are representative of two independent experiments. (**E**) The area occupied by the cells was measured from the microscopy images and plotted as relative cell area. Data represents mean with SEM, derived from six images analyzed for each experimental group from two independent experiments, where at least 3 cells were measured in each image (N ≥ 18). *p* values were determined using one-way ANOVA; *, *p* < 0.05; ns, not significant, for results compared with buffer-treated cells incubated with SOZ particles.

Engagement of phagocytic receptors triggers downstream signaling and tyrosine phosphorylation of crucial signaling proteins, provoking actin cytoskeleton rearrangement and uptake of opsonized targets [4,8,10,12,13]. To examine the effects of toxin-provoked cAMP intoxication on opsonophagocytic signaling-mediated actin cytoskeleton remodeling and on changes in cell shape/morphology, we pretreated THP-1 cells with buffer, CyaA, or ET and subsequently added SOZ particles. THP-1 cells incubated with unopsonized zymosan particles were taken as negative control. The cells were then fixed, permeabilized, and stained for F-actin with phalloidin, followed by microscopy. As shown in Figure 4A, THP-1 monocytes treated with unopsonized zymosan particles showed no cell spreading, and actin cytoskeleton was observed as a uniform ring along the periphery of the cell. In contrast, incubation of cells with SOZ promoted cell spreading and formation of characteristic actin-rich membrane protrusions called filopodia (Figure 4B). However, SOZ-induced actin cytoskeleton rearrangement and formation of filopodia was inhibited upon preincubation of phagocytes with CyaA (Figure 4C), but not with ET (Figure 4D). Furthermore, similar trends were observed when the area occupied by the cells was measured from the microscopy images (Figure 4E) representing the differential effects of toxin provoked cAMP signaling on SOZ-induced cell spreading and filopodia formation.

## 3. Discussion

Phagocytosis is a multistep process that involves receptor-mediated interaction and recognition of phagocytic targets, actin cytoskeleton remodeling facilitating uptake, phagosomal membrane remodeling, phagosome maturation, and finally, the clearance of the ingested particles. A variety of signal transduction pathways contribute to each step of the carefully orchestrated and tightly controlled cellular phagocytic process [1,2]. Particle uptake and clearance are known to be reduced or inhibited in phagocytic cells where one or more of the crucial signaling proteins are missing/mutated [8,10,13,14]. These signaling proteins/mediators exist in a hierarchical sequence in a particular signaling cascade and their activities are influenced by crosstalk, making them part of a larger dynamic signaling network that controls the bactericidal action of phagocytes. The activity of many of these signaling mediators is controlled by phosphorylation/dephosphorylation, spatial localization, and (re)distribution in the cytoplasm in the course of uptake and clearance of opsonized targets. At different stages of phagocytosis, Syk, Pyk2, and Vav are localized to the phagosomes and drive the signaling events that lead to actin remodeling, which plays a crucial role in particle uptake [8,10,14]. Furthermore, a well-regulated, cellular adenylate cyclase-driven accumulation of cAMP at the nascent phagosome has been observed during phagosome formation, having a direct effect on actin cytoskeleton rearrangement during phagocytosis [46,47,48]. However, the direct link between the cellular adenylate cyclase driven regulated cAMP accumulation and the initiation of tyrosine kinase cascades by Src family kinases in the context of myeloid phagocytes is currently unknown. In contrast, bacterial toxin-provoked accumulation of cellular cAMP at supraphysiological levels in phagocytes inhibits bactericidal activities and phagocyte functions [16,19,34,35,36,49].

In this study, we demonstrated that CyaA-mediated cAMP elevation, but not ET-mediated cAMP elevation to comparable overall levels, triggers signaling events that inhibit the uptake of opsonized targets (Figure 1). CyaA-triggered cAMP signaling completely inhibited opsonin-induced phosphorylation of crucial signaling mediators Syk, Vav, and Pyk2 downstream of the phagocytic receptors. In contrast, ET-triggered cAMP signaling partially inhibited Syk and Vav phosphorylation and had no significant effect on Pyk2 phosphorylation (Figure 2). Indeed, CyaA toxin action instantly leads to the formation of a cAMP pool in the submembranous region of the cytoplasm, which may directly modulate the signaling cascades in the local/specific target region on the cytosolic side of the membrane [50,51]. Further indirect evidence is provided by microscopy experiments where Syk [8], Pyk2 [10], and Vav [13,14] have been shown to be recruited/accumulated in the submembranous region at sites of active uptake of opsonized particles, which is the cytoplasmic subdomain where membrane proximal CyaA-mediated cAMP accumulation occurs. For example, the activity of the L-type Ca^2+^ channel Ca_v_1.2 is influenced by intrinsic AC activity through cAMP-mediated activation of PKA, which is counterbalanced by the activity of the phosphatase PP2A, and all of these enzymes assemble into a membrane-proximal multi-molecular signaling complex [52]. It was recently shown that CyaA-mediated cAMP signaling activates Src homology domain 2 containing protein tyrosine phosphatase (SHP-1), thus suppressing the expression of inducible nitric oxide synthase (iNOS) in macrophages [34]. SHP-1 activation also contributes to suppression of oxidative burst in neutrophils [53] and enables suppression of bactericidal activities of phagocytes [26,29]. However, the causality between dephosphorylation of Syk, Vav, and Pyk2 and CyaA-mediated SHP-1 activation remains to be established. Indeed, the postulated biological function of ET is to slow down the progression of lethal toxin-provoked apoptosis in infected macrophages. This would strike a critical balance between cell death and survival, thereby facilitating dissemination of *B. anthracis* to the lymph nodes, from where the replicating bacteria can disseminate systemically [31]. Importantly, during infection the ET acts in synergy with the lethal toxin to inhibit macrophage activation and functions.

cAMP signaling can be highly compartmentalized. It is heavily influenced by the cell specific expression of PKA isozymes, the expression and specific subcellular localization of A-kinase anchoring proteins (AKAPs; anchoring PKA close to specific targets), the distribution and localization of Epac, and the localization and regulation of phosphodiesterases (regulating local amounts of cyclic nucleotides) [47,51,54,55,56,57]. Indeed, localized compartmentalization of cAMP signaling has been described in cardiac myocytes [51,55], T-cells [50], and neurons [52], but remains to be fully elucidated in myeloid phagocytes. In macrophages, the cAMP-mediated effects exhibit differential involvement of Epac and PKA in suppression of various phagocyte functions [19]. Recently, it was shown that CyaA toxin action, but not the action of ET, lead to compartmentalized accumulation of cAMP in T-cells, thus inhibiting the accumulation of critical regulatory proteins and directly affecting the formation of immune synapse [50], whereas ET has been shown to induce the phosphorylation of the cAMP response element-binding protein (CREB) and activation of gene transcription in T-cells [39]. We can speculate that the localized accumulation of cAMP in phagocytes is able to efficiently elicit cAMP signaling in that specific cytoplasmic subdomain, thus efficiently influencing the tyrosine phosphorylation of signaling mediators.

ET-mediated cAMP accumulation, with a gradient radiating from the perinuclear region, was shown to provoke IL-6 secretion and inhibit LPS-induced TNF-α secretion in bone marrow derived-macrophages [45], paralyze essential natural killer cell functions [58], and impair type-IIA secreted phospholipase A2 synthesis in alveolar macrophages [40]. Some of these effects can be reversed by the pretreatment of cells with adefovir, an inhibitor of AC activity [40,45]. These studies show that ET-mediated cAMP accumulation has direct effects on various leukocyte functions other than uptake and clearance of opsonized targets in human monocytic cells. Furthermore, ET-mediated cAMP elevation has been reported to reduce the uptake of non-opsonized targets (*B. anthracis* Ames spores and fluorescent *E. coli* particles) in differentiated human macrophages [20]. However, the receptors involved, the phagocytic machinery employed, and the associated signaling cascades activated in opsonic- and non-opsonic phagocytosis are different. These differences are further influenced by the molecular characteristics of the targets involved, the cellular receptors engaged, and the types of phagocytes involved [2,7,59].

## 4. Conclusions

Taken together, this study shows that the spatial distribution of toxin-provoked cAMP intoxication in phagocytes governs the inhibition of particle uptake and phosphorylation/activation of important signaling proteins. This reveals that mere accumulation of supraphysiological amounts of cAMP in the cytoplasm is not sufficient for effective modulation of some of the signaling pathways involved in opsonophagocytic particle uptake and that appropriate spatial distribution of cAMP is key to effective inhibition of opsonophagocytic uptake by the adenylyl cyclase enzyme toxins.

## 5. Materials and Methods

### 5.1. Reagents and Antibodies

RPMI 1640 and DMEM were obtained from Sigma-Aldrich, St. Louis, MO. Antibodies against p-PYK2 (sc-293142), p-Vav (sc-16408-R), and Vav (sc-17831) were obtained from Santa Cruz Biotechnology Inc., Dallas, TX. Anti-phosphotyrosine (11-263-C100) and anti-Syk (11-376-C100) antibodies were purchased from Exbio, Vestec, Czech Republic. Anti-Pyk2 antibody (P3902) was purchased from Sigma-Aldrich, St. Louis, MO. Antibody against p-Syk (MAB6459) was purchased from R&D systems, Minneapolis, MN. Rabbit polyclonal anti-cAMP antibody for competitive ELISA was obtained from GenScript, Piscataway, NJ. Horseradish peroxidase (HRP)-conjugated anti-mouse and anti-rabbit IgG antibodies were purchased from GE Healthcare, Piscataway, NJ. HBSS buffer (10 mM HEPES, pH 7.4, 140 mM NaCl, 5 mM KCl) complemented with 2 mM CaCl_2_, 2 mM MgCl_2_, 1% (*w*/*v*) glucose, and 1% (*v*/*v*) fetal calf serum (FCS)) was prepared for flow cytometry experiments. Zymosan A was purchased from Sigma-Aldrich, St. Louis, MO. For labeling, zymosan at 10 mg/ml was incubated with 50 ug/ml of fluorescein isothiocyanate (FITC; Sigma-Aldrich, St. Louis, MO) in 300 mM sodium bicarbonate buffer (pH 9.2) with overnight rotation at 4 °C. Unbound FITC was removed by extensive washing with HBSS buffer, and FITC labeled zymosan (FITC-zymosan) was stored at −20 °C.

### 5.2. Toxins

CyaA was produced in the presence of activating acyltransferase CyaC in *E. coli* strain XL1-Blue (Stratgene, La Jolla, CA). The protein was chromatographically purified by ion exchange chromatography on DEAE-Sepharose and hydrophobic chromatography on Phenyl-Sepharose, from which endotoxin was removed by repeated washing of the toxin-bound resin with 60% isopropanol [26,60]. The toxin preparation contained less than 0.1 EU/µg of protein as determined by Limulus Amebocyte Lysate assay (QCL-1000, Lonza, Walkersville, MD) and was stored in 50 mM Tris (pH 8.0), 8 M urea, and 2 mM CaCl_2_ buffer at ‒20 °C. Recombinant EF and PA from *Bacillus anthracis* were purchased from List Biological Laboratories, Inc., Campbell, CA.

### 5.3. Cell Culture

THP-1 human monocytic cells (ATCC TIB-202) were obtained from the American Type Culture Collection (ATCC, Manassas, VA) and tested for the absence of mycoplasma contamination. The cells were cultured in RPMI 1640 supplemented with 10% fetal calf serum and antibiotic antimycotic solution. The cells were transferred to DMEM with 10% FCS, without antibiotic antimycotic solution for toxin treatment, inhibitor treatment, and signal transduction experiments.

### 5.4. cAMP Assay

6 × 10^5^ THP-1 cells were incubated with CyaA (150 ng/ml; 0.85 nM) or ET (625 ng/ml; 7.04 nM EF + 2500 ng/ml; 30.24 nM PA) for different time durations in 200 µl of DMEM with 10% FCS and no antibiotics at 37 °C. The reaction was stopped by lysing the pellet by addition of 0.2% Tween-20 in 50 mM HCl. The lysates were boiled for 10 min at 100 °C, neutralized by addition of 150 mM unbuffered imidazole, and cellular cAMP levels were measured using competitive ELISA, as described elsewhere [61]. cAMP concentrations were normalized to the total protein content, which was determined using a micro-BCA protein assay kit (Bio-Rad, Rockford, IL).

### 5.5. Signal Transduction Experiments

Zymosan particles were opsonized by incubating the particles with 50% human serum for 30 min at 37 °C. The particles were then washed and dissolved in DMEM with FCS before use for treatment of cells (100 µg of zymosan particles per 10^6^ cells). To investigate the effects of toxin-provoked cAMP intoxication on opsonophagocytic signaling, 3 × 10^6^ THP-1 cells were preincubated with CyaA (150 ng/ml; 0.85 nM) for 30 min or ET (625 ng/ml; 7.04 nM EF + 2500 ng/ml; 30.24 nM PA) for 6 h, respectively (based on cAMP ELISA experiments to have similar cAMP intoxication levels, *cf*. Figure 1A,B). The cells were then treated with 300 µg of serum opsonized or unopsonized zymosan as positive and negative controls for 30 min, respectively.

Cells were preincubated for 6 h with an AC inhibitor (adefovir dipivoxil) [45] at a final concentration of 10 µM in DMEM with 10% FCS and no antibiotics at 37 °C. The experiments were then continued as mentioned above in the constant presence of the inhibitory drug.

### 5.6. Immunoblotting

Cellular lysates were analyzed by immunoblotting as previously described [26]. Briefly, experiments were stopped at respective time points by lysing the cells with Triton X lysis buffer (50 mM Tris-HCl (pH 7.4), 150 mM NaCl, 1 mM Na_3_VO_4_, 10 mM NaF, 0.3% SDS, 1% Triton X 100, and EDTA-free protease inhibitor cocktail (Roche Diagnostics GmbH, Mannheim, Germany)). The proteins were separated using SDS-PAGE and transferred onto polyvinylidene difluoride membranes. The blots were probed with indicated primary antibodies, followed by incubation with horseradish peroxidase-conjugated secondary antibody and developed using SuperSignal West Femto maximum sensitivity substrate (Thermo Scientific, Rockford, IL, USA). The chemiluminescent signal was recorded using a G: Box Chemi gel documentation system (Syngene, Frederick, MD, USA). The immunoblots were analyzed using AIDA Image Analysis software (Raytest, Straubenhardt, Germany), and an AIDA 2D Densitometry module was used to evaluate the intensity of the chemiluminescent signal. The background-subtracted density of the bands was measured for each individual band from immunoblots probed by phospho-specific antibodies and normalized by the corresponding density of bands from immunoblots probed by antibodies recognizing the total form of the signaling protein concerned. The values obtained from the positive controls (Buffer + SOZ or Adefovir + SOZ) were set at 100%.

### 5.7. Flow Cytometry

2 × 10^5^ THP-1 cells were incubated with CyaA or ET as mentioned above, washed with HBSS buffer and treated with serum opsonized FITC-zymosan particles (10 µg of zymosan particles per 10^5^ cells) for 30 min at 37 °C or 4 °C in HBSS buffer. Toxin and opsonized FITC-zymosan particles were simultaneously added to the cells in some experiments. THP-1 cells were preincubated with adefovir dipivoxil for 6 h prior to treatment with toxin and subsequent incubation with opsonized FITC-zymosan in some experiments. The reaction was stopped by addition of 3 volumes of chilled HBSS buffer, the cells were centrifuged and resuspended in 100 µl of chilled HBSS buffer. Live THP-1 cells were selected by performing the flow cytometric analysis in the presence of Hoechst 33258 (1 µg/ml), and the cells were further selected (and FITC-zymosan particles excluded) using gating on a bi-dimensional cytogram based on signals from forward scatter and side scatter. Data were analyzed using FlowJo software (Tree Star, Ashland, OR). The number of cells that incorporated fluorescent zymosan particles was determined on a fluorescence histogram. Phagocytic activity was expressed as phagocytic index (PhI) [62], calculated using the formula:

(1) $$PhI=\frac{\%PhT}{\%PhT0}$$

where PhT represents FITC positive cells after 30 min of incubation at 37 °C (fluorescence contributed by both bound and internalized zymosan particles), and PhT0 represents the FITC positive cells in the corresponding control incubated at 4 °C (fluorescence contributed by bound particles alone).

### 5.8. Immunofluorescence and Confocal Microscopy

To visualize the actin cytoskeleton and the cell morphology, 3 × 10^6^ THP-1 cells were seeded on cover slips (2 h) in DMEM with 10% FCS and no antibiotics. The cells were then treated at 37 °C with CyaA (150 ng/ml; 0.85 nM) for 30 min or ET (625 ng/ml EF; 7.04 nM + 2500 ng/ml; 30.24 nM PA) for 6 h to obtain similar overall levels of cAMP intoxication. The toxin treated or untreated cells were incubated with serum opsonized zymosan or unopsonized zymosan particles (100 µg of zymosan per 10^6^ cells) for 30 min at 37 °C. The cells were washed twice with warm PBS (37 °C), fixed by incubation with 3.7% paraformaldehyde for 15 min, and permeabilized by 0.1% Triton in PBS. The cover slips were blocked with 5% BSA in PBS for 1 h and stained with TRITC conjugated phalloidin dissolved in 2% BSA (1:50) in the presence of 1 μg/ml of DAPI. Finally, the coverslips were washed three times with PBS for five minutes each, rinsed once with distilled water, and mounted in the inverted position on a microscopic slide in Vectashield mounting medium. Immunofluorescent images were obtained using an Olympus FV-1000 confocal microscope (Olympus Corporation, Tokyo, Japan). Reconstructed z stack projections (average intensity) of the images were used for measuring the cell area. The cell periphery was traced using freehand selection tools in ImageJ, the area enclosed was measured using the measure function and plotted as relative cell area, where the area of Zymosan treated cells was set as 1.0 (negative control).

### 5.9. Statistical Analysis

One-way analysis of variance (ANOVA) was used to perform statistical analysis followed by Tukey’s test or Dunnett’s test for post hoc analysis. A *p* value less than 0.05 was considered statistically significant. All statistical analyses were performed with Prism (version 6) software (GraphPad, La Jolla, CA). The axis was split in some graphs to facilitate the accurate representation of the trends.
